# Non-Caucasian Race/Ethnicity Predisposes to Unfavorable Stage and Grade at Upper Tract Urothelial Carcinoma Diagnosis

**DOI:** 10.1007/s40615-025-02285-0

**Published:** 2025-01-09

**Authors:** Francesco Di Bello, Mario de Angelis, Carolin Siech, Natali Rodriguez Peñaranda, Zhe Tian, Jordan A. Goyal, Claudia Collà Ruvolo, Gianluigi Califano, Massimiliano Creta, Fred Saad, Shahrokh F. Shariat, Alberto Briganti, Felix K. H. Chun, Salvatore Micali, Nicola Longo, Pierre I. Karakiewicz

**Affiliations:** 1 Cancer Prognostics and Health Outcomes Unit, Division of Urology, University of Montréal Health Center, Montréal, Québec Canada; 2 Department of Neurosciences, Science of Reproduction and Odontostomatology, University of Naples Federico II, Via Pansini, 80131 Naples, Italy; 3 Vita-Salute San Raffaele University, Milan, Italy; 4 Division of Experimental Oncology/Unit of Urology, URI, Urological Research Institute, IRCCS San Raffaele Scientific Institute, Milan, Italy; 5 Department of Urology, University Hospital, Goethe University Frankfurt, Frankfurt Am Main, Germany; 6 Department of Urology, Ospedale Policlinico E Nuovo Ospedale Civile S. Agostino Estense Modena, University of Modena and Reggio Emilia, Modena, Italy; 7 Department of Urology, Comprehensive Cancer Center, Medical University of Vienna, Vienna, Austria; 8 Department of Urology, Weill Cornell Medical College, New York, NY USA; 9 Department of Urology, University of Texas Southwestern Medical Center, Dallas, TX USA; 10 Hourani Center for Applied Scientific Research, Al-Ahliyya Amman University, Al-Salt, Amman Jordan

**Keywords:** UTUC, SEER, Racial disparities, Stage-specific, Asian

## Abstract

**Objective:**

To test whether race/ethnicity affects stage or grade distribution at upper tract urothelial carcinoma (UTUC) diagnosis.

**Methods:**

Within the Surveillance, Epidemiology, and End Results (SEER) database 2004–2020, UTUC patients were identified. Multivariable logistic regression models tested for the association between race/ethnicity and stage as well as grade at diagnosis according to renal pelvis vs. ureteral origin. Stage at presentation was defined as (i) metastatic stage (T_any_N_any_M_1_) and (ii) advanced stage (locoregional or metastatic (T_3-4_N_0-2_M_0-1_)).

**Results:**

Of 14,384 UTUC patients, 8926 (62%) were renal pelvis. Of renal pelvis UTUC patients, 7064 (79%) were Caucasian, 797 (9%) Hispanic, 623 (7%) Asian Pacific Islander (API), and 442 (5%) African American (AA). Relative to Caucasians, API (odd ratio (OR) 1.85, 95% confidence interval (CI) 1.44–2.37, *p* < 0.001) and AA (OR 1.37, 95% CI 1.02–1.83, *p* = 0.03) patients more frequently harbored metastatic stage. APIs also more frequently harbored advanced (OR 1.33, 95% CI 1.11–1.60, *p* = 0.002) stage and high (OR 1.33, 95% CI 1.07–1.67, *p* = 0.01) grade. Of 5458 (38%) ureteral UTUC patients, 4360 (80%) were Caucasian, 362 (7%) Hispanic, 509 (9%) API, and 227 (4%) AA. Relative to Caucasians, Hispanic (OR 1.45, 95% CI 1.01–2.05, *p* = 0.03) patients more frequently harbored metastatic stage. APIs also more frequently harbored advanced stage (OR 1.22, 95% CI 1.02–1.47, *p* = 0.03) and high (OR 1.94, 95% CI 1.49–2.55, *p* < 0.001) grade.

**Conclusions:**

Race/ethnicity other than Caucasian, such as API and Hispanic, may predispose to higher odds of metastatic (T_any_N_any_M_1_) or advanced (T_3-4_N_0-2_M_0-1_) stages as well as to higher grade at initial diagnosis.

## Introduction

Race/ethnicity other than Caucasian previously appeared to affect various urothelial cancer characteristics [1–3]. Specifically, historical non-Caucasian upper tract urothelial carcinoma (UTUC) patients appeared to harbor less favorable stage and grade at presentation [4–7]. For instance, Matsumoto et al. observed a high prevalence of non-organ confined and high-grade disease in Asiatic RNU patients within 20 academic centers across America, Asia, and Europe [7]. However, this report analyzed only 2163 RNU patients from 1987 to 2008 [7]. Thus, it is unknown whether this disadvantage has been eliminated in contemporary UTUC patients. We addressed this knowledge gap and tested the association between race/ethnicity and stage and grade at UTUC initial diagnosis. Specifically, we hypothesized that in non-Caucasian UTUC patients, namely Asian Pacific Islander (API), Hispanic, and African American (AA), the rate of metastatic stage, advanced stage, and high grade is higher than in their Caucasian counterparts. To test these hypotheses, we relied on the most contemporary Surveillance, Epidemiology, and End Results (SEER) database 2004–2020.

## Materials and Methods

### Study Population

Within the SEER database (2004–2020), we identified patients ≥ 18 years old with histologically confirmed urothelial carcinoma of the renal pelvis and ureteral (International Classification of Disease (ICD-10) site code C65, C66). Patients with unknown vital status, unknown SEER stage, as well as grade were excluded [8]. Autopsy- or death-certificate-only cases were also not included [9]. These selection criteria yielded 14,384 assessable patients.

### Variables Definition

Included patients were stratified according to race/ethnicity (Caucasian, Hispanic, API, and AA). The same stratification was first applied to renal pelvis UTUC patients and, subsequently, to ureteral UTUC patients. Patient variables consisted of age at diagnosis (years), sex (male vs. female), stage (metastatic stage (T_any_N_any_M_1_) and advanced stage (locoregional or metastatic, T_3–4_N_0–2_M_0–1_)), and grade (high vs. low).

### Statistical Analyses

**Fig. 1:**
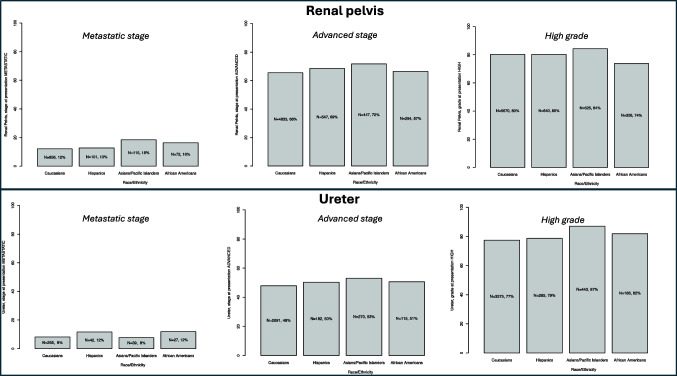
Stage distribution of newly diagnosed patients with renal pelvis and ureteral upper tract urothelial carcinoma (UTUC) according to race/ethnicity (Caucasian vs. Hispanics vs. Asian Pacific Islanders vs. African American), within the SEER database (2004–2020)

**Fig. 2:**
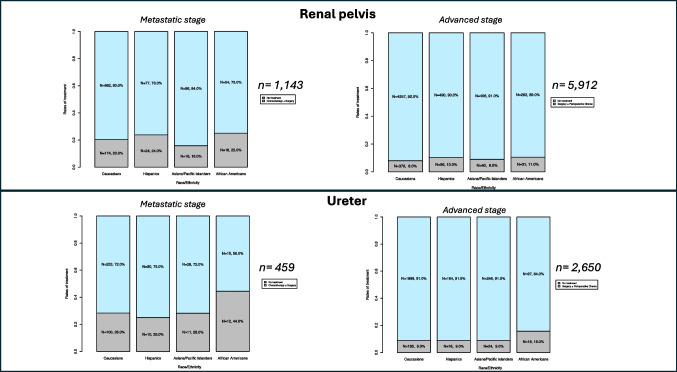
Treatment patterns distribution of newly diagnosed patients with renal pelvis and ureteral upper tract urothelial carcinoma (UTUC) according to race/ethnicity (Caucasian vs. Hispanics vs. Asian Pacific Islanders vs. African American), within the SEER database (2004–2020). The *n* refers to the total sample after the exclusion of patients with no available treatment options

Four analytical steps were completed. First, baseline patient characteristics were tabulated (Table 1). Descriptive statistics included medians and interquartile ranges (IQR) for continuously coded variables and frequencies and proportions for categorical variables. The Kruskal–Wallis rank sum test examined the statistical significance of medians’ differences for continuous variables. Pearson’s chi-square test assessed the statistical significance in proportions’ differences for categorical variables. Thereafter, separate multivariable logistic regression models (LRMs) addressed three specific endpoints (Table 2). The first endpoint consisted of rates of metastatic (T_any_N_any_M_1_) stage at initial diagnosis (Fig. 1). The second endpoint consisted of rates of advanced (T_3–4_N_0–2_M_0–1_) stage at initial diagnosis (Fig. 1). Finally, the third specific endpoint consisted of the presence of high grade at initial diagnosis (Fig. 1). Fourth, subgroup analyses with separate multivariable LRMs predicting access to treatment were completed (Table 3). The first subgroup analysis focused on access to treatment, defined as chemotherapy ± cytoreductive surgery, within metastatic (T_any_N_any_M_1_) stage patients (Fig. 2) [10]. The second subgroup analysis focused on access to treatment, defined as surgery ± perioperative chemotherapy, within advanced (T_3–4_N_0–2_M_0–1_) stage patients (Fig. 2) [11–13]. All four analytical steps were first applied to renal pelvis UTUC patients and then were re-applied to their ureteral UTUC counterparts. All tests were two-sided, with a significance level set at *p* < 0.05. In all statistical analyses, the R software environment for statistical computing and graphics (R version 4.1.3, R Foundation for Statical Computing, Vienna Austria; http://www.r-project.org/) was used [14].

**Table 1:** Descriptive characteristics of 14,384 upper tract urothelial carcinoma (UTUC) patients stratified according to race/ethnicity (Caucasian vs. Hispanics vs. Asian Pacific Islanders vs. African American), within the SEER database (2004–2020)

	Upper tract urothelial carcinoma (*N* = 14,384)							
	Renal pelvis, *N* = 8926 (62%)				Ureter, *N* = 5458 (38%)			
	Caucasian, *N* = 7064 (79%)^a^	Hispanic, *N* = 797 (9%)^a^	API, *N* = 623 (7%)^a^	African American, *N* = 442 (5%)^a^	Caucasian, *N* = 4360 (80%)^a^	Hispanic, *N* = 362 (7%)^a^	API, *N* = 509 (9%)^a^	African American, *N* = 227 (4%)^a^
Age at diagnosis	72 (64, 78)	70 (62, 77)	71 (63, 77)	66 (58, 75)	73 (66, 78)	71 (64, 77)	72 (65, 77)	70 (64, 76)
Sex								
Male	4294 (61%)	486 (61%)	344 (55%)	243 (55%)	2840 (65%)	219 (60%)	288 (57%)	116 (51%)
Female	2770 (39%)	311 (39%)	279 (45%)	199 (45%)	1520 (35%)	143 (40%)	221 (43%)	111 (49%)
Grade								
High grade	5670 (80%)	640 (80%)	525 (84%)	326 (74%)	3375 (77%)	285 (79%)	443 (87%)	186 (82%)
Stage at presentation								
Localized	2431 (34%)	250 (31%)	176 (28%)	148 (33%)	2269 (52%)	180 (50%)	239 (47%)	112 (49%)
Regional	3777 (53%)	446 (56%)	332 (53%)	222 (50%)	1736 (40%)	140 (39%)	231 (45%)	88 (39%)
Distant	856 (12%)	101 (13%)	115 (18%)	72 (16%)	355 (8%)	42 (12%)	39 (8%)	27 (12%)

Abbreviations: *APIs* Asian Pacific Islanders, *IQR* interquartile range

^a^Median (IQR); *n* (%)

**Table 2:** Multivariable logistic regression models predicting metastatic stage (T_any_N_any_M_1_), advanced stage (T_3–4_N_0–2_M_0–1_ plus T_any_N_any_M_1_), and the presence of high grade at initial diagnosis in 14,384 upper tract urothelial carcinoma (UTUC) patients according to race/ethnicity (Caucasian vs. Hispanics vs. Asian Pacific Islanders vs. African American)

Race/ethnicity	Multivariable logistic regression model predicting:					
	Metastatic stage (T_any_N_any_M_1_)		Advanced stage (T_3–4_N_0–2_M_0–1_)		High grade	
	Renal pelvis	Ureter	Renal pelvis	Ureter	Renal pelvis	Ureter
	OR (95% CI)^a^	OR (95% CI)^a^	OR (95% CI)^a^	OR (95% CI)^a^	OR (95% CI)^a^	OR (95% CI)^a^
Hispanics vs. Caucasians	1.14 (0.89, 1.45)	1.45 (1.01, 2.01)*	1.15 (0.985, 1.35)	1.10 (0.89, 1.36)	1.03 (0.86, 1.25)	1.08 (0.83, 1.41)
APIs vs. Caucasians	1.85 (1.44, 2.37)**	0.91 (0.63, 1.27)	1.33 (1.11, 1.60)*	1.22 (1.02, 1.47)*	1.33 (1.07, 1.67)*	1.94 (1.49, 2.55)**
African Americans vs. Caucasians	1.37 (1.02, 1.83)*	1.45 (0.94, 2.17)	1.05 (0.85, 1.29)	1.12 (0.85, 1.46)	0.74 (0.59, 0.92)*	1.30 (0.93, 1.87)

Abbreviations: *APIs* Asian Pacific Islanders, *CI*, confidence intervals, *OR* odds ratio

^*^Statistical significance ≤ 0.03

^**^Statistical significance ≤ 0.001

^a^Adjusted for age and sex

**Table 3:** Multivariable logistic regression models predicting access to treatment at metastatic stage (T_any_N_any_M_1_) and at advanced stage (T_3–4_N_0–2_M_0–1_ plus T_any_N_any_M_1_) in 14,352 upper tract urothelial carcinoma (UTUC) patients according to race/ethnicity (Caucasian vs. Hispanics vs. Asian Pacific Islanders vs. African American)

Race/ethnicity	Multivariable logistic regression model predicting access to treatment in:			
	Metastatic stage (T_any_N_any_M_1_)°		Advanced stage (T_3–4_N_0–2_M_0–1_)°°	
	Renal pelvis	Ureter	Renal pelvis	Ureter
	OR (95% CI)^a^	OR (95% CI)^a^	OR (95% CI)^a^	OR (95% CI)^a^
Hispanics vs. Caucasians	0.71 (0.43, 1.19)	1.21 (0.58, 2.70)	0.68 (0.51, 0.93)*	0.97 (0.58, 1.72)
APIs vs. Caucasians	1.15 (0.68, 2.04)	1.02 (0.50, 2.24)	0.83 (0.59, 1.19)	0.99 (0.64, 1.59)
African Americans vs. Caucasians	0.58 (0.33, 1.07)	0.44 (0.19, 0.99)*	0.57 (0.94, 0.96)**	0.49 (0.29, 0.86)*

Abbreviations: *APIs* Asian Pacific Islanders, *CI* confidence intervals, *OR* odds ratio

^*^Statistical significance ≤ 0.04

^**^Statistical significance ≤ 0.001

°Treatment for metastatic stage means chemotherapy ± cytoreductive surgery

°°Treatment for advanced stage means surgery ± perioperative chemotherapy

^a^Adjusted for age and sex

## Results

### Descriptive Characteristics of Renal Pelvis UTUC Patients

Within the SEER database 2004–2020, we identified 14,384 UTUC patients. Of those, 8926 (62%) harbored renal pelvis UTUC and 5458 (38%) harbored ureteral UTUC. Of renal pelvis UTUC patients, 7064 (79%) were Caucasian, 797 (9%) Hispanic, 623 (7%) API, and 442 (5%) AA (Table 1). Metastatic (T_any_N_any_M_1_) stage at presentation was identified in 18% of APIs, 16% of AAs, 13% of Hispanics, and 12% of Caucasians. Advanced (T_3–4_N_0–2_M_0–1_) stage at presentation was identified in 72% of APIs, 69% of Hispanics, 67% of AAs, and 66% of Caucasians. High grade at presentation was identified in 84% of APIs, 74% of AAs, 80% of Hispanics, and 80% of Caucasians. Of metastatic renal pelvis UTUC patients, 25% of AAs, 24% of Hispanics, 20% of Caucasians, and 16% of APIs were not treated with chemotherapy ± cytoreductive surgery. Of advanced stage renal pelvis UTUC patients, 11% of AAs, 10% of Hispanics, 9% of APIs, and 8% of Caucasians were not treated with surgery ± perioperative chemotherapy.

### Renal Pelvis UTUC: Race/Ethnicity vs. Stage and Grade at Presentation

In multivariable LRMs, relative to Caucasians, API race/ethnicity (odds ratio (OR) 1.85, 95% confidence interval (CI) 1.44–2.37, *p* < 0.001) and AA race/ethnicity (OR 1.37, 95% CI 1.02–1.83, *p* = 0.03) independently predicted higher rate of metastatic stage (T_any_N_any_M_1_). Moreover, API race/ethnicity (OR 1.33, 95% CI 1.11–1.60, *p* = 0.002), but neither AA nor Hispanic, independently predicted a higher rate of advanced stage (T_3–4_N_0–2_M_0–1_). Finally, API race/ethnicity (OR 1.33, 95% CI 1.07–1.67, *p* = 0.01), but neither AA nor Hispanic, also independently predicted a higher rate of high grade.

### Renal Pelvis UTUC: Race/Ethnicity vs. Access to Treatment

In multivariable LRMs, relative to Caucasians, Hispanic race/ethnicity (OR 0.68, 95% CI 0.51–0.93, *p* = 0.01) and AA race/ethnicity (OR 0.57, 95% CI 0.94–0.96, *p* < 0.001), but not API, exhibited lower access to treatment at advanced stage (T_3–4_N_0–2_M_0–1_). No race/ethnicity group exhibited lower access to treatment at the metastatic stage (T_any_N_any_M_1_).

### Descriptive Characteristics of Ureteral UTUC Patients

Of 5458 (38%) ureteral UTUC patients, 4360 (80%) were Caucasian, 362 (7%) Hispanic, 509 (9%) API, and 227 (4%) AA (Table 1). Metastatic (T_any_N_any_M_1_) stage at presentation was identified in 12% of Hispanics, 12% of AAs, 8% of APIs, and 8% of Caucasians. Advanced (T_3–4_N_0–2_M_0–1_) stage at presentation was identified in 53% of APIs, 51% of AAs, 50% of Hispanics, and 48% of Caucasians. High grade at presentation was identified in 87% of APIs, 82% of AA, 79% of Hispanics, and 77% of Caucasians. Of metastatic ureteral UTUC patients, 44% of AAs, 28% of APIs, 28% of Caucasians, and 25% of Hispanics were not treated with chemotherapy ± cytoreductive surgery. Of advanced stage ureteral UTUC patients, 16% of AAs, 9% of Hispanics, 9% of APIs, and 9% of Caucasians were not treated with surgery ± perioperative chemotherapy.

### Ureteral UTUC: Race/Ethnicity vs. Stage and Grade at Presentation

In multivariable LRMs, relative to Caucasians, Hispanic race/ethnicity (OR 1.45, 95% CI 1.01, 2.05, *p* = 0.03) independently predicted a higher rate of the metastatic stage (T_any_N_any_M_1_). Moreover, API race/ethnicity (OR 1.22, 95% CI 1.02–1.47, *p* = 0.03), but neither AA nor Hispanic, independently predicted a higher rate advanced stage (T_3–4_N_0–2_M_0–1_). Finally, API race/ethnicity (OR 1.94, 95% CI 1.49–2.55, *p* < 0.001), but neither AA nor Hispanic, also independently predicted a higher rate of high grade.

### Ureteral UTUC: Race/Ethnicity vs. Access to Treatment

In multivariable LRMs, relative to Caucasians, AA race/ethnicity invariably exhibited lower access to treatment at metastatic stage (T_any_N_any_M_1_: OR 0.44, 95% CI 0.19–0.99, *p* = 0.04) as well as at advanced stage (T_3–4_N_0–2_M_0–1_: OR 0.49, 95% CI 0.29–0.86, *p* = 0.01).

## Discussion

In historical reports, non-Caucasian race/ethnicity represented a risk factor for more unfavorable stage and grade at UTUC initial diagnosis [7, 15–17]. We tested whether this unfavorable association still applies to most contemporary UTUC patients and made several noteworthy observations.

First, in the current study, we identified 8926 (62%) renal pelvis UTUC patients and 5458 (38%) ureteral UTUC patients. Of both renal pelvis and ureteral patients, the vast majority consisted of Caucasians (79% and 80%). Among renal pelvis UTUC, 21% were non-Caucasian, and of those, Hispanics accounted for 9% of the total population, followed by APIs (7%) and AAs (5%) in that order. Virtually, a very similar distribution was recorded in ureteral UTUC patients, where APIs accounted for 9% of the total population, followed by Hispanics (7%) and AAs (4%) in that order. These observations attest to the rarity of UTUC as a primary tumor. Additionally, they also attest to the even greater rarity of non-Caucasian race/ethnicity among those patients. The observations regarding total count and race/ethnicity proportions are in agreement with previous albeit and substantially more historical SEER report (1988–2007) that also addressed UTUC patients [5]. Unfortunately, no other studies addressed UTUC based on other large-scale databases. In consequence, other direct comparisons cannot be made.

Second, we recorded important stage and grade differences at initial diagnosis according to race/ethnicity groups. In renal pelvis UTUC patients, race/ethnicity other than Caucasian, namely API (OR 1.85) and AA (OR 1.37), independently predicted a higher rate of metastatic (T_any_N_any_M_1_) stage (*p* ≤ 0.03). Additionally, API (OR 1.33) also independently predicted a higher rate of advanced stage that included metastatic (T_any_N_any_M_1_) and locoregional (T_3–4_N_0–2_M_0_) stages (*p* = 0.002). Regarding ureteral UTUC, Hispanic (OR 1.45) and API (OR 1.22) race/ethnicity, respectively, independently predicted a higher rate of metastatic (T_any_N_any_M_1_) and advanced (T_3–4_N_0–2_M_0–1_) stage (*p* = 0.03). Taken together those observations indicate that non-Caucasian race/ethnicity is frequently associated with unfavorable stage at presentation. Within the current analysis, the latter was first defined as metastatic stage alone (T_any_N_any_M_1_) and in subsequent analyses was re-assessed using a different definition that combined locoregional and metastatic stages (T_3–4_N_0–2_M_0–1_). In both instances, non-Caucasian race/ethnicity frequently independently predicted a worse outcome. A similar pattern was recorded for high grade at presentation in both renal pelvis and ureteral UTUC.

Third, we also recorded important access to treatment discrepancies according to race/ethnicity groups. Specifically, in renal pelvis UTUC patients, race/ethnicity other than Caucasian, namely AA (OR 0.57) and Hispanic (OR 0.68), exhibited lower access to treatment at advanced stage (T_3–4_N_0–2_M_0–1_). Conversely, in ureteral UTUC patients, AA invariably exhibited lower access to treatment at metastatic stage (OR 0.44) as well as at advanced stage (OR 0.49). Among four race/ethnicity groups, AA virtually exhibited lower access to treatment while not APIs. Thus, it may be postulated that despite APIs having an unfavorable stage and grade at UTUC initial diagnosis, it does not translate into a lower access to treatment. Conversely, AAs exhibited either the unfavorable stage ad initial diagnosis or a lower access to treatment. It may be due to late diagnosis, low chance to receive optimal treatment, or also higher odds to wait longer to be treated [18–20]. Unfortunately, that information is not available in the SEER database. Moreover, especially for UTUC, such results cannot be compared with other studies such other studies do not exist. However, despite AA accounting for almost 5% of UTUC patients, they should equally be given particular attention with the intent of reducing the access to treatment inequity that may translate into worse survival.

Taken together, these findings indicate that non-Caucasian race/ethnicity remains a risk factor for the examined unfavorable stage and grade definitions at UTUC diagnosis. This observation is worrisome given the fact that historical studies previously reported very similar observations and associations where non-Caucasian patients presented with more unfavorable stage and grade [6, 7, 21]. Ideally, unfavorable stage and grade at diagnosis in non-Caucasian patients, relative to their Caucasian counterparts, would have been eliminated in contemporary UTUC populations. Persistence of unfavorable association between race/ethnicity and tumor characteristics (stage and grade) suggests that efforts aimed at sensitizing the urologic community about the need for greater attention to the potential presence of UTUC in non-Caucasian patients should not be abandoned but instead should be intensified with the intent to eliminate these race/ethnicity disadvantages that were previously recorded in non-Caucasian patients.

Despite the noteworthy observations, the current study is not devoid of limitations. First and foremost, the current study shares the limitations of all similar studies that were based on the SEER database or other population-based repositories [22, 23] and relied on a retrospective data design [2, 6, 24, 25]. Second, the amount of detail available in the SEER database is limited. Indeed, multivariable adjustment relied on clinically meaningful characteristics available in the SEER database. It is possible that other patient and tumor characteristics that are not available in the current database, such as detailed comorbidities, baseline performance status, and laboratory values, may also play a role in the association between race/ethnicity and unfavorable stage and grade at UTUC initial diagnosis as well as in the association between race/ethnicity and access to treatment [26, 27]. Moreover, household income is not available within the SEER repository. However, their unavailability in the SEER database renders such analyses impossible. Last but not least, central pathology review, such as usually applied to prospective randomized trials, is also not available.

## Conclusion

Race/ethnicity other than Caucasian, such as API and Hispanic, may predispose to higher odds of metastatic (T_any_N_any_M_1_) or advanced (T_3–4_N_0–2_M_0–1_) stages as well as to higher grade at initial diagnosis. However, further studies that may overcome the limitations of the current database are needed to better understand the discrepancies among race/ethnicity enlightened within the current study.

## Data Availability

All analyses and their reporting followed the SEER reporting guidelines. The dataset supporting the conclusions of this article is available in the SEER Incidence Data, 1975–2020 repository, https://seer.cancer.gov/data/. The specific datasets generated during and/or analyzed during the current study are available from the corresponding author on reasonable request.
